# CANCROX: a cross-species cancer therapy database

**DOI:** 10.1093/database/baz044

**Published:** 2019-04-25

**Authors:** Paulo Muniz de Ávila, Diego Cesar Valente e Silva, Paulo Cesar de Melo Bernardo, Ramon Gustavo Teodoro Marques da Silva, Ana Lúcia Fachin, Mozart Marins, Edilson Carlos Caritá

**Affiliations:** 1 Biotechnology Unit, University of Ribeirão Preto, Av. Costábile Romano, Ribeirão Preto, SP, Brazil; 2 Federal Institute of Education, Science and Technology of South of Minas Gerais; 3 Federal Institute Of Education, Science and Technology of São Paulo; 4 Medicine School, University of Ribeirão Preto, Av. Costábile Romano, Ribeirão Preto, SP, Brazil; 5 Center for Exact, Natural and Technological Sciences, University of Ribeirão Preto, Ribeirão Preto SP, Brazil

## Abstract

Cancer comprises a set of more than 200 diseases resulting from the uncontrolled growth of cells that invade tissues and organs, which can spread to other regions of the body. The types of cancer found in humans are also described in animal models, a fact that has raised the interest of the scientific community in comparative oncology studies. In this study, bioinformatics tools were used to implement a computational model that uses text mining and natural language processing to construct a reference database that relates human and canine genes potentially associated with cancer, defining genetic pathways and information about cancer and cancer therapies. The CANCROX reference database was constructed by processing the scientific literature and lists more than 1300 drugs and therapies used to treat cancer, in addition to over 10 000 combinations of these drugs, including 40 types of cancer. A user-friendly interface was developed that enables researchers to search for different types of information about therapies, drug combinations, genes and types of cancer. In addition, data visualization tools allow to explore and relate different drugs and therapies for the treatment of cancer, providing information for groups studying animal models, in this case the dog, as well as groups studying cancer in humans.

## Introduction

The amount of information available in the biomedical literature is enormous, and every year the number of new publications grows substantially. According to Larsen and von Ins (1), estimated annual growth rates range from 2.2–9%. Based on these estimates, more than 10 million articles will be published every year and the currently available literature will double in less than a decade. An analysis of the PubMed database (2) reveals expressive numbers, with the existence of >24 million citations and >24 000 indexed journals. Among these citations, abstracts are available for more than 12 million publications and 13 million possess links to the full text, with both an abstract and a full text link being available for 9.8 million (3). Clearly, this amount of information produced by the scientific community is too vast for any researcher to read and assimilate the whole volume produced and published. The simple process of searching for information already produces a number of results that are difficult to analyze. It is therefore necessary to develop methods that permit to retrieve and make available information, inferring new knowledge and contributing to the progress in biomedical research, especially that related to groups of malignant diseases such as cancer. Known as a lethal disease, cancer caused the death of 8.2 million people worldwide in 2012 (4). The number of new cases is estimated to increase by ~70% in the next two decades. Cancer is therefore one of the most important fields of study in the biomedical sciences. A PubMed search using ‘cancer’ as a search term returned >3.2 million publications and that number continues to grow. This large volume of information is almost exclusively made available in text form, which allows the processing and transformation of these data into more structured formats using computational tools such as text mining (TM). TM tools can be applied in different areas of knowledge, including the important area of cancer research. Considering the complexity involved in the study of cancer, animal models have become valuable tools for studying the biology and genetics of human cancers, as well as for the pre-clinical investigation of therapies and disease prevention (5). Many animal species develop cancer spontaneously and represent an interesting model for research, especially because some of these species had their genetic sequences mapped, a fact that increases the capacity for comparisons between these species and humans (*Homo sapiens*). Among these species, the dog (*Canis lupus familiaris*) has been shown to be a good animal model (6, 7). The relationship between humans and dogs is ancient and dates back at least 15000 years, or even up to 100 000 years. Dogs were first domesticated from the gray wolf in East Asia (7, 8) and evolved through a mutually beneficial relationship with humans, sharing the habitat and food sources. As a consequence of this evolution, many modern dog breeds exhibit a high prevalence of specific diseases, including cancer, blindness, epilepsy and heart diseases. These diseases are also observed in the human population, and their clinical manifestations are similar in the two species. Thus, the results of studies conducted on dogs may be applied to humans and viceversa. Within this context, we developed the CANCROX tool that employs TM and natural language processing (NLP) techniques to establish the relationship between human cancers and similar cancers in dogs in order to identify treatment and prevention strategies. The CANCROX database contains important information about cancer treatment, cancer prevention and associated genes and a wide range of drugs and their combinations.

## Materials and methods

### CANCROX

**Figure 1:**
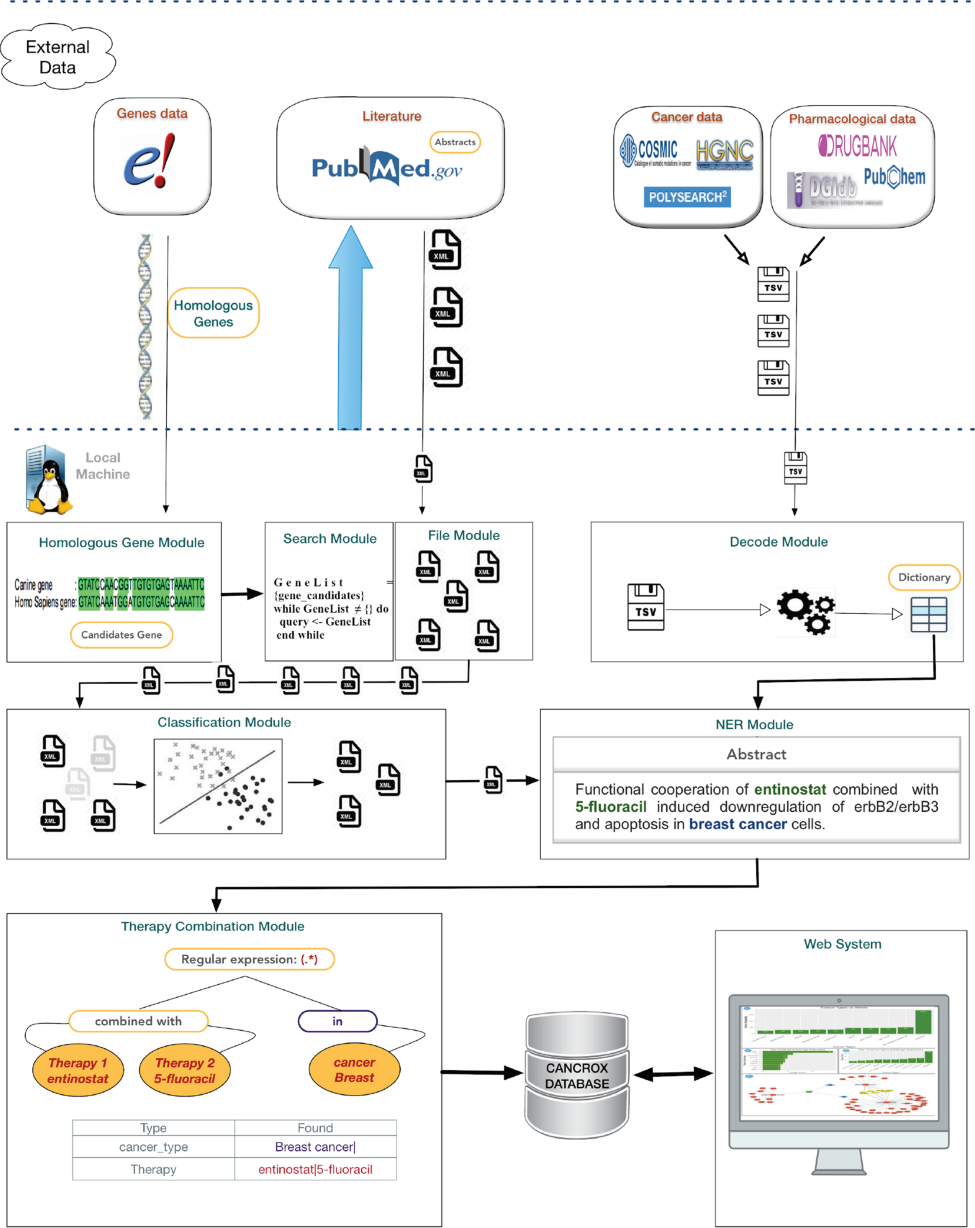
Architecture of the CANCROX database. The pipeline starts with the download of external databases and pre-processing, which consists of organizing the different data patterns in structured database tables. Next, the pre-processed data are sent to the learning algorithms of the machine (where articles are classified as ‘positive results’ and ‘negative results’) and articles with ‘positive results’ are submitted to the NER module, where therapies, drugs and types of cancer are recognized. In the final step of this processing phase, the combination of therapies is identified. The first tier of the tool grants access to external databases to obtain information about genes, drugs and scientific texts. Processing and persistence of the data occur in the second tier. The third tier is responsible for providing mechanisms of data access, i.e. permitting visualization of the processed data.

The CANCROX tool is able to perform TM and NLP analysis in thousands and even millions of scientific articles, constructing a reference database of similar genes between humans and dogs associated with cancer, hereinafter referred to as candidate genes. In addition to candidate genes, the types of cancer associated with these genes, therapies and drugs and their different combinations are identified and stored in this database. Figure 1 illustrates the architecture of the CANCROX tool, which was developed as a three-tier application, each consisting of well-defined modules.

### External databases and data pre-processing

The first step of our pipeline consists of the automated download of eight external databases divided into four information categories: cancer-associated genes, human genes and other species, biomedical literature and pharmacological data. These databases permit the implementation of article queries, elaboration of cancer therapy dictionaries and alignment of genetic sequences between humans and dogs. The data on human genes and other species permit to determine similar genes between humans and dogs. Sequencing of the dog genome demonstrated conserved synteny between the canine and human genome for 94% of the segments. Among the 19000 canine genes reported, 14200 are 1-1 orthologs of humans and dogs (9), i.e. these genes share a common ancestor and have the same functions in the species. For download of the candidate genes (14200), we used the R statistical language (10) and the biomaRt package, which provides an interface for the BioMart data warehouse (11), a system that allows to manage the large volumes of data produced by the Ensembl project (12). Information such as the ID that identifies the human and canine genes, the percentage of similarity between genes (0–100%) and a description of the genes were obtained and used for processing of the candidate gene list. The candidate genes are obtained by cross-referencing similar genes and genes with somatic mutations, i.e. it is necessary to identify those genes that effectively undergo somatic mutation among all genes that are similar between humans and dogs. The database of the Catalog of Somatic Mutation in Cancer (COMIC) project was used to determine the set of human genes that undergo somatic mutation. Version v72 of this database contains a total of 572 genes with somatic mutations identified through the medical literature and analyzed by experts. In addition to providing a curated database, the project provides access to information via the FTP protocol for automatic updates (13). Implementation of a script in PERL language was necessary for mapping the human genes described in the COMIC database and the similar human and dog genes obtained by the Ensembl project. This script enabled the identification of agreement between unique gene identifiers (geneID) of the Entrez Gene project (14) used as IDs of genes in the COMIC database and unique identifiers of the Ensembl project *ensembl_gene_id* used as IDs of genes that are similar between humans and dogs (15). The PERL script used the gene_info.gz database available for download at ftp://ftp.ncbi.nlm.nih.gov/gene/DATA/. After processing of the data, the symbols representing the corresponding genes were identified and mapped. In addition, the identifiers of the Human Genome Organization (HUGO), a public database that ensures that each gene is given only one unique approved symbol (16), and of the Mendelian Inheritance in Man (OMIM) (17), a database that lists all human diseases with a genetic component, were mapped and associated. This mapping permitted to identify 477 genes with a percent similarity $\geq $75%. These are candidate genes, and this list can be used to construct search queries for accessing and retrieving biomedical literature. The construction of the search query is automated using a script in R language. To obtain the best search results, a table of synonyms was constructed from the processing of the gene_info.gz database (ftp://ftp.ncbi.nlm.nih.gov/gene/DATA/). In addition to synonyms, the unique identifiers of the HUGO and OMIM projects were added to the table. For example, to retrieve all publications related to the BRCA1 gene, the query *(((((((((((((BRACA1) OR BRCAI) OR BRCC1) OR BROVCA1) OR FANCS) OR IRIS) OR PNCA4) OR PPP1R53) OR PSCP) OR RNF53) OR HGNC: 1100) OR MIM:113705) OR Ensembl: ENSG00000012048) OR Ensembl:ENSCAFG00000014600)* is submitted to the PubMed library. PubMed returns a set of files in XML format containing the abstract and title of the articles. On 18 October 2017, the above search query returned 10 030 articles. The whole process is repeated for the other 476 genes of the candidate list. The information about human drugs was obtained by processing and analyzing the main available databases on drugs and substances: DrugBank (18), PubChem (19), DGLdb (20), PharmKb (21) and ChemSpider (22). In general, the first tier of the architecture searches for information in external databases and processes this information by implementing a list of candidate genes and dictionaries. The latter serves as a support tool during the TM and NLP processes.

### Processing

Processing occurs after the download of the external data and pre-processing of the data obtained. The processing modules are responsible for executing algorithms that effectively extract knowledge from the biomedical literature, i.e. the identification of drugs and therapies, types of cancer associated with genes and combination of drugs and therapies occurs in these modules. In addition, these modules are responsible for classifying scientific articles, i.e. separating texts that report cases of ‘success’ from those reporting negative results for a given cancer treatment. For this purpose, a text classifier (classification module), a recognition module of therapy names supported by dictionaries and a set of 27 regular expression rules (NER module) and an identifier of therapy and drug combinations (therapy combination module) were designed and implemented. These three modules are discussed below.

#### Classification module:

In this step, each article of a set of documents to be processed is analyzed. The objective is to define whether this article reports a case of success or failure of the treatment used. For this purpose, a random forest classifier (23) was implemented using the Scikit-learn library (24). To train this classifier, a set of 4000 scientific articles were annotated manually. Annotation consists of indicating whether a given article belongs to class 0 or class 1, with 0 corresponding to an article reporting treatment failure and 1 to an article reporting treatment success. Before executing the random forest classifier, pre-processing is first performed. This consists of tokenization, removal of stopwords, stemming, case normalization and spelling normalization. Next, a term-document matrix is constructed considering *n*-grams of 3 items (trigrams). This matrix serves as the entry for the classification algorithms.

#### NER module:

This module is responsible for recognizing the name of a drug or therapy in the titles and abstract of scientific articles that were not eliminated in the classification step, i.e. articles that report treatment ‘success’. For this purpose, the NER module executes three different tasks: pre-processing, algorithm training and name recognition. Pre-processing consists of the execution of sentence segmentation and tokenization algorithms. Standard implementations of these algorithms are available in the Apache Open NLP library (25). The training step consists of generating a model from an annotated test base. The process of annotation comprises the identification (annotation) of the name of drugs in the text of scientific articles. Approximately 10000 articles were annotated for construction of the model. The step of name recognition, also known as named entity recognition (NER), consists of receiving a text and returning a list of words identified according to an implemented model. The method used in this study is based on maximum entropy modeling (26) implemented in the Open NLP library. In the present study, the drugs are almost always identified. However, one problem is that, in addition to drugs and therapies, other words that have some relation with drugs are also identified, for example, the word ‘inhibitor’, the expression ‘ $P\leq 0.001$’ and several others. To minimize this problem of false positives, a dictionary containing the main words and expressions identified during the process was elaborated using regular expressions. The aim of this approach is to help eliminate most ‘junk’. A simple regular expression that eliminates a large number of false positives is the following: [w]^{+}[>|=|>=][d |.]^{+}. This expression eliminates any word that starts with a letter, has an equal-to or greater-than-equal-to sign and ends with a numerical sequence, such as the word ‘$P\leq 0.001$’.

**Figure 2:**
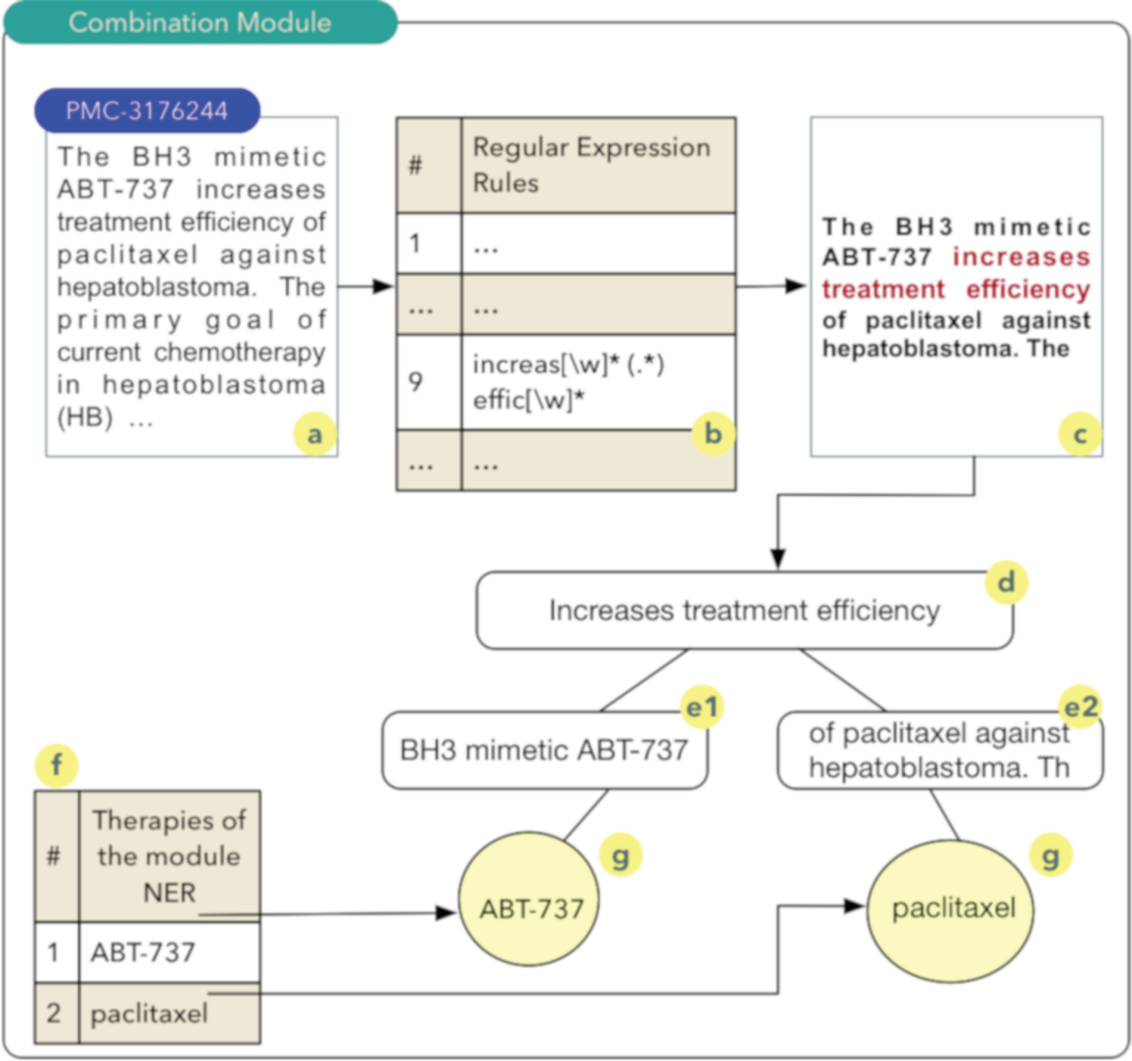
Combination module.

#### Drug and therapy combination module:

The aim of the ‘drug and therapy combination’ module is to identify combinations of drugs and therapies for cancer treatment. Studies exploring this approach started in the early 1950s, when a group of drugs was used for the treatment of cancer and other diseases (27). A computational technique known as regular expressions was used for the construction of this module. Regular expressions permit to identify standards or rules in scientific texts that indicate a high probability of the combination of drugs or therapies. Thus, a set of 27 rules were implemented. Once these rules are obtained, a series of steps are executed as shown in Figure 2.

It should be noted that the process shown in Figure 2 occurs for all articles comprising the database of scientific abstracts. The algorithm starts by reading the abstract (2a), and each of the rules are sought in the text of the abstract (2b). In the example shown in Figure 2, rule number 9 was identified. Thus, the text fragment composed of the five words before the rule found and the five words after the rule are recovered (2c). The part highlighted in red in Figure 2c shows the text fragment that was identified by rule 9. This text fragment, defined as control phrase, is stored in a computational structure known as tree. The control phrase is stored in the root node (2d), i.e. the parent node of the tree. Next, the five words that precede the control phrase are stored in a child node on the left (e1) of the root node. The same process occurs for the five words located immediately to the right of the control phrase (e2). The list of drugs (2f) identified during processing in the NER module is used to search and identify drugs present in the left and right nodes of the tree. The therapies identified during this search process have a high probability of being combinations and are stored in the database. In this example, the combination of the drugs ABT-737 and paclitaxel (28) was identified (2g).

## Results

Computationally, the CANCROX database is formed by 10 related tables that store different data collected in the literature. Table 1 shows a high-level view of the database developed in this study.

**Table 1:** Biological databases used in this study

Type of information in the database	Number of entries in database
Cancer genes	477
Oncogenes	355
Tumor suppressor genes	122
Pharmacological data and therapies	11 688
Drugs and substance processed	1308
DrugBank	656
PubChem	451
ChemSpider	104
New drugs and substances	97
Combination of drugs and substances	10 380

**Table 2:** Metrics of the random forest classifier

Classifier	Precision	Recall	*F*-score
Random forest	91.8%	85.39%	88.47%

**Table 3:** Metrics of the NER

Classifier	Precision	Recall	*F*-score
Max entropy (A)	71.20%	66.39%	68.71%
Max entropy + regular expression (B)	87.12%	86.61%	86.86%

**Figure 3:**
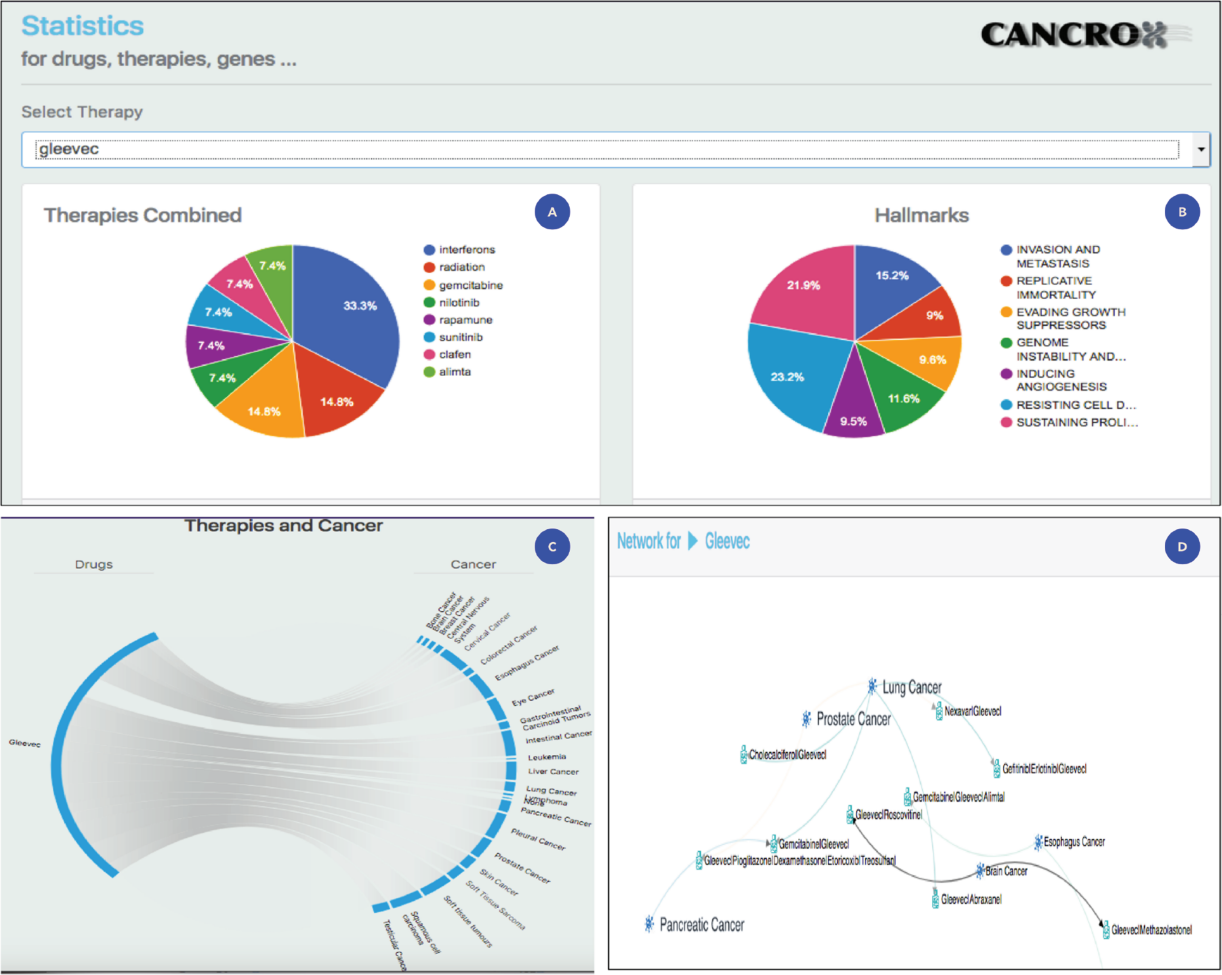
Statistical analysis of the drug Gleevec or imatinib mesylate. (A) Main therapies combined with Gleevec for the treatment of different types of cancer, including interferons, radiation, gemcitabine and nilotinib, among others. (B) Data obtained by accessing the databases of the CHAT project (29) (http://chat.lionproject.net./). According to the literature, Gleevec is classified as ‘evasion and metastasis’. (C) Graphical representation of the 20 types of cancer treated with the drug. (D) A snipped image of the relationship network between Gleevec and its respective therapy combinations, associated with the respective types of cancer.

It is important to point out that the data shown in Table 1 were obtained by the analysis of more than 338 000 articles. From this processing, 1308 drugs were identified, in addition to substances and therapies. In addition, a total of 10 380 drug combinations were obtained and persisted in the database. To increase the reliability of the data, a process known as cross-referencing was applied to establish a relationship of the drugs identified with the CANCROX tool with other external databases such as ChemSpider and PubChem. During this process, 1211 drugs and substances were identified and mapped to the external databases. Interestingly, 97 drugs and substances were not located in these databases. These included experimental drugs, drugs synthesized from an analogous drug and new drugs not yet properly stored in these databases. This fact is interesting and confirms the efficiency of the NER algorithm, ~87% accuracy, in identifying and extracting drugs from the literature. The Web interface offers a search mechanism for 477 genes of the 10 380 drug combinations and 40 types of cancer.

### Evaluation metrics for the classifiers and NER

Evaluation metrics permit to verify the efficiency of the classification algorithm (random forest) and of the algorithm for identifying therapies and drugs (NER). The following data were obtained by training of the random forest algorithm using 4000 articles that were annotated with value 0 for those reporting treatment failure and value 1 for those reporting treatment success. This number corresponds to 2.95% of the 338445 articles available for classification. The training parameters of the algorithm were defined as $k\textrm {-fold}=10$ for cross-validation and construct validation of 300 trees in the random forest algorithm. The algorithm was executed, and the metrics obtained are shown in Table 2.

As can be seen in Table 3, two models were implemented to NER. This was necessary after the low performance obtained with algorithm (A). Interestingly, the precision of 71.2% is the result of a large number of false positives, i.e. the model identifies correctly drugs and therapies, but a series of other words are recognized erroneously. Model (B) considerably improved the final result. The combination of the maximum entropy algorithm (34) implemented in the Apache OpenNLP library with regular expressions improved the final result by 22.35%. The combination of techniques permitted considerable elimination of noise in the final result.

### Case study: imatinib mesylate

Imatinib mesylate, also known as Gleevec and Glivec, is a tyrosine kinase inhibitor. According to the National Cancer Institute site, this drug is used for the treatment of the following cancers: acute lymphoblastic leukemia, chronic eosinophilic leukemia, chronic myelogenous leukemia, dermatofibrosarcoma protuberans, gastrointestinal stromal tumor, myelodysplastic/myeloproliferative neoplasms and systemic mastocytosis. Thus, a set of seven cancers are treated with this drug. Compared to the data of the National Cancer Institute, the CANCROX tool identified 20 cancer categories and 45 therapy combinations, numbers larger than those reported by the National Cancer Institute. A consolidated view of these data is shown in Figure 3.

Figure 3 shows the statistical analysis of the drug Gleevec. It is important to note that the tool permits to analyze any of the 1308 treatments (drugs and therapies) that make up the database. Using the statistical analysis tool, researchers can verify the main combinations of therapies applied to different types of cancer. In addition, it is possible to access the title and abstract referring to each therapy combination identified by the tool.

### Comparison with other databases

For comparison and evaluation of the CANCROX database in terms of drug combination, the following databases were selected in the literature: Antifungal Synergistic Drug Combination Database (ASDCD) (30) and Drug Combination Database (DCDB) (31). The number of databases that explore drug combinations is limited. The ASDCD lists a total of 1225 drug combinations and 105 individual drugs obtained from 12 000 references of the medical literature. This database is specialized on drug combinations used for the treatment of fungal infections. However, some of these drugs are also used to treat different types of cancer. Comparison between the ASDCD and CANCROX databases identified 35 individual drugs present in both databases, i.e. drugs used to treat fungi and to combat cancer. A total of 92 drug combinations were identified simultaneously in the two databases. Another database that uses the approach of drug combinations is DCDB. This database possesses a collection of 1363 combined drugs and 904 individual drugs. Compared to the CANCROX database, the number of individual drugs is 31% lower. Since that database was last updated in 2014, this difference of 404 individual drugs can be explained by the research and discovery of new compounds during the period after the last update of the DCDB. Similar to the approach adopted in the present study, ~14% of the studies on drug combinations report ‘failure’ of the experiments. This number is close to that obtained during the classification phase of the articles of the present study, in which ~17% of the articles were identified as reporting ‘failure’ of the treatment employed.

## Conclusions

CANCROX is the first tool that focuses on the implementation of a reference database of similar human and canine genes associated with cancer. This database provides researchers using this animal model with opportunities to access and analyze a set of 477 genes associated with more than 40 types of cancer and more than 10 000 combinations of drugs and therapies for this disease. In this version, CANCROX focuses on the canine model. However, the architecture of the tool permits the use of other models as a reference, for example, the mouse (32) and zebrafish (33). The CANCROX database contains important information about cancer treatment, prevention of cancer and associated genes and drugs and therapies and their different combination, thus providing data for groups studying animal models, in this case the dog, as well as groups studying cancer in humans. The CANCROX database is therefore expected to become a platform of consolidated information that helps the scientific community in this important field of research, i.e. cancer. The initial planning of this work foresees the updating of the CANCROX database in 2 years.

*Conflict of interest*. None declared.

## Notes

**Database URL:** http://cancrox.gmb.bio.br/
